# Confocal Microscopy and Image Analysis Indicates a Region-Specific Relation between Active Caspases and Cytoplasm in Ejaculated and Epididymal Sperm

**DOI:** 10.1371/journal.pone.0035477

**Published:** 2012-04-17

**Authors:** Susana García Vazquez, Andrés Aragón Martínez, Juan Carlos Flores-Alonso

**Affiliations:** 1 Laboratorio de Biología de la Reproducción, Facultad de Medicina Veterinaria y Zootecnia, Universidad Autónoma del Estado de México, El Cerrillo, Toluca, México; 2 Departamento de Biología de la Reproducción, Universidad Autónoma Metropolitana Iztapalapa, México, Distrito Federal, México; 3 Laboratorio de Biología de la Reproducción, Centro de Investigación Biomédica de Oriente, Hospital General de Zona #5, Metepec, Instituto Mexicano del Seguro Social, Metepec, Puebla, México; Tufts University, United States of America

## Abstract

Previously, it was suggested a relation between the presence of apoptosis markers with cytoplasm in mammalian sperm. In this work, flow cytometry, confocal microscopy and image analysis were used to analyze the relationship between active caspase-3 and -7 and intracellular esterases expression in ejaculated or epididymal ram sperm. Sperm obtained from ejaculates from the caput, corpus, or cauda of the epididymis were treated with an inhibitor of active caspase-3 and -7 and a marker of cytoplasmic esterases. Additionally, ejaculated sperm were incubated for one, two, or three hours before evaluation for active caspases. Sperm subpopulations positive for active caspases and/or intracellular esterases were detected by flow cytometry and confocal microscopy; however, image analysis of confocal images showed that the correlation between active caspases and cytoplasmic esterases in sperm is region-specific. Lower values of Spearman correlation coefficients were found when whole sperm or head sperm was analyzed; however, a high correlation was observed for midpiece sperm. Incubation of sperm for two or three hours promoted the autoactivation of caspases. It has been suggested that the presence of apoptotic markers in sperm are related with a process of abortive apoptosis and with errors during spermiogenesis. Our results permit us suggest that the origin of the relationship between active caspases and cytoplasmic esterases is due to differentiation errors occurring during spermiogenesis because the percentages of sperm with active caspases are not different in the caput, corpus, or cauda of the epididymis. In this study we demonstrate that existing sperm subpopulations can express active caspases and intracellular esterases and that the correlation between these molecules is high in midpiece sperm.

## Introduction

Somatic [1] and testicular germ cells [2], [3] die by apoptosis. Research has demonstrated that in the testicular germ cell of rats, some markers of apoptosis are restricted to residual bodies [4], suggesting that the apoptotic machinery moves the germ cells from a cytoplasmic state to a “without cytoplasm state” during differentiation toward mature sperm; however, apoptosis markers such as caspases are detected in sperm of different species [5]–[9]. Caspases are proteases related with apoptosis that have been detected in most nucleated metazoan cells studied [10] and are the active form of the zimogen expressed natively [2], [11]. Activation of caspase-3 and -7 is indicative that the cell is committed to die [2]. In this sense, it is intriguing that active caspases are detected in sperm of different species, including man [6], pig [7], and ram [8], and stallion [9]. The physiological role of active caspases in sperm is not known. Further, the origin of active caspases in ejaculated sperm is not known. Early Weng et al. [12] suggested a relationship between spermatic cytoplasm and active caspases in ejaculated sperm, and Sakkas et al. [13] coined the term abortive apoptosis for those ejaculated sperm that present molecular apoptosis markers. Related with this, a intense fluorescent signal caspases-induced in cytoplasmic droplets was reported for human sperm [14]; and recently we observed a similar phenomenon in boar [15] and ram sperm (observation not published).

Because of this, we hypothesize that the abnormal presence of cytoplasm in sperm could be related to the presence of active caspases.

Calcein is a non fluorescent membrane permeable used to detect viable cell [1], [16]. This dye can freely diffuse inside the cell, where it is transformed in a polar molecule that cannot cross back the intact membrane, due this property calcein has been used as marker to detect spermatic cytoplasm movement in membrane fusion experiments [17].

On the other hand, active caspases are detected immediately when sperm are ejaculated; this clearly indicates that sperm with active caspases are in the male reproductive tract, although it is not know where the caspases were activated. During traverse for different regions of epididymis the sperm undergo a series of changes called maturation, one of these changes involve elimination of cytoplasmic droplet [18]. At present it is not known whether the presence of sperm carrying active caspases is distinct in different regions of epididymis.

Caspases in sperm are prone to be activated [14]; however, some reports indicated that activation of caspases is not easy in sperm incubated [13], [5], [9]. Due sperm with active caspases has been related with infertility and that assisted reproduction techniques involves processing sperm *in vitro* it is possible that caspases are activated during such process. In this study we analyzed a) the relationship between spermatic cytoplasm and active caspases in ejaculated sperm, b) whether the presence of sperm with active caspases change during epididymal transit, and c) whether the presence of active caspases is related with incubation time.

## Materials and Methods

### Sample collection

All experiments were performed using neat semen. The rams were 1.5 years-of-age and weighed 65–80 kg. Subject animals were kept at the Facultad de Medicina Veterinaria y Zootecnia under uniform nutritional conditions. Semen samples were collected from four mature mexican creole rams. Ejaculates were obtained from each ram every second day. At least five ejaculates were obtained from each animal. With the aim of minimizing the rams' stress, the technician in charge of obtaining the ejaculates was always the same. Rams were human killed according to Norma Oficial Mexicana NOM-033-ZOO-1995 and epididymides were obtained. The protocol of this work was revised by Bioethics Committee of Facultad de Medicina Veterinaria y Zootecnia the nine of October of 2009 and approval letter (s/n) was received.

In order to achieve the objective of this work, three experiments were conducted. For the first, active caspases-3 and -7, and cytoplasm were detected in ejaculated sperm by flow cytometry and image analysis of confocal microscopy. For the second experiment, ejaculated sperm were incubated for one, two, or three hours before evaluating the presence of active caspases. For the third experiment, the presence of active caspases-3 and -7, and cytoplasm was evaluated by flow cytometry in sperm from different regions of the epididymis.

### Detection of coexpression of active caspases and intracellular esterases

Active caspases-3 and -7 activity were labeled *in situ* with a fluorescent inhibitor of caspases (FLICA™) using the Image-iT™ LIVE Red Caspase-3 and -7 Detection Kit (Molecular Probes Inc., Eugene, OR, USA) according to the manufacturer's instructions. Briefly, 10 µl of a FLICA working solution was added to 300 µl of sperm and mixed gently. The suspension was incubated for 60 minutes at 37°C and protected from light. After incubation, the samples were stained with an indicator of cytoplasm. In this study, we used calcein as an indicator of cytoplasm. Calcein-acetoxymethylester (calcein AM) is a non-fluorescent membrane-permeable molecule that is reduced to fluorescent calcein by intracellular eesterases [1], [16]. Calcein AM contained in the LIVE/DEAD®; viability/cytotoxicity kit for mammalian cells (Molecular Probes) was used as following: sperm suspension at a concentration of 25 µM of calcein AM was incubated for 15 minutes in the dark at room temperature prior to flow cytometry analysis. To demonstrate FLICA specificity, we first checked the absence of fluorescence in semen samples before addition of FLICA (negative control). Before detection of caspases with FLICA, active caspases were induced *de novo* by adding sodium nitroprusside (Sigma, St Louis, MO, USA) at a final concentration of 400 µM during one hour [7] to semen samples where “endogenous” active caspases were blocked by addition of Z-DEVD-FMK (Sigma), a cell-permeable inhibitor of caspase-3, -6, -7, -8, and -10 (inhibitor control). Aliquotes of the same semen samples were treated only with sodium nitroprusside as above (positive control). Rationale of the previous was that semen samples only treated with sodium nitroprosside should present a higher fluorescence than those where active caspases were first blocked with Z-DEVD-FMK, due “endogenous” and *de novo* active caspases are detected.

The volume of all samples was adjusted to 500 µl with TNE buffer (0.01 M Tris-HCl, 0.15 M NaCl, 1 mM EDTA, pH 7.4) prior to flow cytometry analysis. A FACScan flow cytometer (Becton Dickinson, Immunocytometry Systems, San Jose, CA, USA) equipped with an argon laser (488 nm) was used to evaluate the sperm parameters. A live gate was used in the FSC and SSC parameters to exclude aggregates and debris, thereby, restricting data acquisition to an almost pure population of sperm. List mode data of 10,000 events were collected for each sample using CellQuest 3.3 software (BDIS) in a Power Mac G4 with Mac OS 9. Percentages of sperm positives to active caspases or cytoplasmic esterases were obtained with WinList 3.0 software (Verity Software House, Inc, Topsham, Maine) in a PC with windows XP.

The left epididymis was used to obtain sperm from the caput, corpus and cauda. For one animal, the right epididymis was selected because the left epididymis presented with cysts, without inflammation or other pathological signs. The caput, corpus, or cauda of the epididymis was placed in a petri case with 10 ml of PBS buffer (pH 7.4), minced with scissors, and incubated for 20 min at 35°C. Sperm were filtered with a nylon mesh of 100 µm in a sterile tube of polypropylene. Detection of active caspases and cytoplasmic esterases was done as specified previously.

### Detection of active caspases after different time of incubation

Aliquots of ejaculated sperm were incubated in HTF medium (200 mM sucrose, 50 mM NaCl, 18.6 mM sodium lactate, 21 mM HEPES, 10 mM KCl, 2.8 mM glucose, 0.4 mM MgSO_4_, 0.3 mM sodium pyruvate, 0.3 mM K_2_HPO_4_, 1.5 IU/mL penicillin, and 1.5 mg/mL streptomycin, pH 6.4) (SIGMA) at 37°C under 5% CO_2_ in air during one, two or three hours before evaluated for active caspases. Detection of active caspases was done as specified above.

### Confocal microscopy analysis

To identify the location of caspase-3 and -7 and cytoplasmic esterases in sperm, a smear was made for each semen sample. The cells were examined under a confocal microscope E-800 (Nikon, Yokohama, Japan). Micrographs of sperm coexpressing cytoplasmic and active caspases-3 and -7 were captured with EZ-C1 2.30 software (Nikon).

Almost one image of sperm coexpressing active caspases and intracellular esterases was obtained for each slide (i. e., channel red and green, respectively). Confocal images were digitally processed to obtain the relation between values of pixels in green and red channel. Images were processed with ImageJ 1.42q software [19] and PSC colocalization plugin according with the procedure of French et al., [20] Briefly, confocal images were opened in ImageJ software with the ICS_Opener pluggin and decomposed in red (FLICA, active caspase-3 and-7), green (calcein, intracellular esterases), and blue channels of eight bytes each, and a mask with only the sperm cell inside was made with the aid of the blue channel. Then, the mask was overlayed on channels red and green. In this way, only pixels inside the mask were analyzed. The correlation between pixels forming the sperm in red and green channels was obtained with the PSC colocalization plugin [20] for the entire sperm cell or for specific sperm regions.

### Statistics

All results are expressed as mean ± SEM. Normality of percentages of sperm with active caspases was corroborated graphically in different experiments by checking residuals. An ANOVA for repeated measures was performed to compare percentages of ejaculated sperm with active caspases after different incubation periods, and a one way ANOVA was conducted to analyze the percentages of sperm with active caspases in different regions of epididymides; the Tukey test was used as a post hoc test. Relationships among sperm subpopulations (for flow cytometry analysis) or between pixels of red (active caspase-3 and -7) and green (cytoplasmic esterases) channels of confocal mycroscopy images were analyzed by Spearman correlation. All analysis were performed with the R 2.9.0 software [21] using a macbook with Mac OSX 10.6.2. Results were considered significant at the p<0.05 level.

## Results

**Figure 1:**
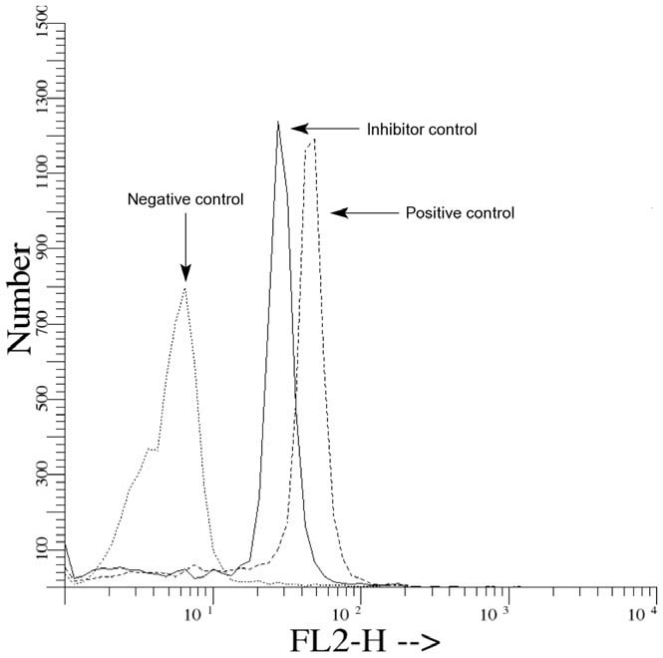
Representative histograms of controls for the active caspases in ejaculated sperm. Negative control, without FLICA. Positive control, sodium nitroprusside before add FLICA. Inhibitor control, “endogenous” active caspases were blocked with Z-DEVD-FMK, then active caspases were induced *de novo* by addition of sodium nitroprosside and detected with FLICA.

**Figure 2:**
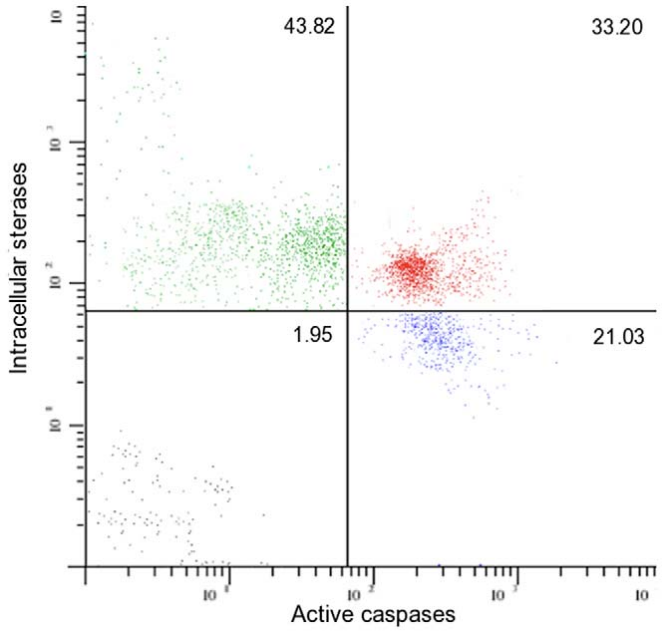
Flow cytometry dotplot showing sperm subpopulations of an ejaculate. Upper left quadrant, sperm expressing intracellular esterases and negatives to active caspases. Upper right quadrant, sperm coexpressing intracellular esterases and active caspases. Lower right quadrant, sperm expressing active caspases and without intracellular esterases. Lower left quadrant, sperm without intracellular esterases nor active caspases. Numbers inside quadrants indicate percentage for each sperm subpopulation.

**Figure 3:**
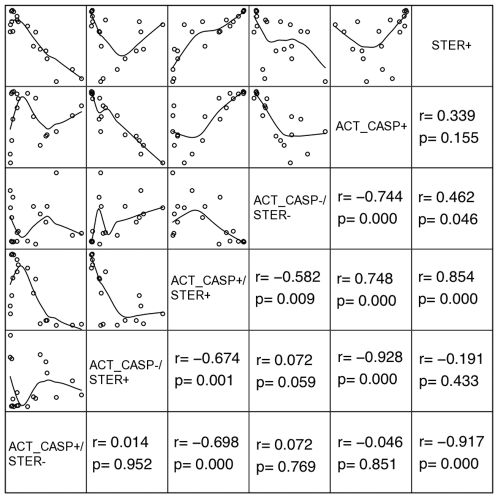
Correlations among distinct sperm subpopulations. Matrix of bivariate plots and Spearman correlation values among percentages of ejaculated sperm subpopulations expressing active caspase-3 and -7 and/or intracellular esterases obtained from flow cytometry analysis. Diagonal upper panel shows bivariate plots of different sperm subpopulations. Inner lines in bivariate plots indicate a general tendency of relation between points. Diagonal lower panel indicates Spearman r and p-value for each plot in a mirror form. Legends on diagonal: ACT_CASP+/STER−, percentage or sperm positives to active caspase-3 and -7 and negatives to cytoplasmic esterases; ACT_CASP−/STER+, percentage of sperm negatives to active caspase-3 and -7 and positives to cytoplamic esterases; ACT_CASP+/STER+, percentage of sperm positives to active caspase-3 and 7 and positives to cytoplasmic esterases; ACT_CASP−/STER−, percentage of sperm negatives to active caspase-3 and-7; ACT_CASP+, percentage total of sperm positives to active caspase-3 and -7 (ACT_CASP+/STER+ plus ACT_CAS+/STER−); STER+, percentage total of sperm positives to cytoplasmic esterases (ACT_CASP−/STER+ plus ACT_CASP+/STER+).

FLICA stained active caspases in sperm, but when a nonfluorescent inhibitor of active caspases was added before FLICA, that stain diminished (Fig. 1). Based on staining properties detected by flow cytometry, different sperm subpopulations in ejaculates were identified. The mean total percentage (i.e., sperm subpopulation with only expression of active caspases plus sperm subpopulation that expressed active caspases and intracellular esterases) of sperm with expression of active caspase-3 and -7 was 70.1±6.0, whereas the mean percentage of sperm that coexpressed intracellular esterases and active caspase-3 and -7 was 46.8±8.3. The mean total percentage (i. e., sperm subpopulation with only expression of intracellular esterases plus sperm subpopulation that expressed active caspases and cytoplasmic esterases) of sperm that expressed intracellular esterases was 65.0±6.3. The mean percentage of the sperm subpopulation that expressed only intracellular esterases was 18.2±4 (Fig. 2). A matrix of correlations among different sperm subpopulations is shown in Fig. 3. A negative relationship was observed for total percentages of sperm with intracellular esterases and percentages of sperm positive for active caspases but negative for intracellular esterases. Also, a strong negative relationship was obtained for the total percentage of sperm with active caspases and the percentage of sperm negative for active caspases but positive for cytoplasmic esterases; a positive relation was observed for the percentage of total sperm positive for intracellular esterases and the percentage of sperm positive for active caspases and positive for cytoplasmic esterases (Fig. 3).

**Figure 4:**
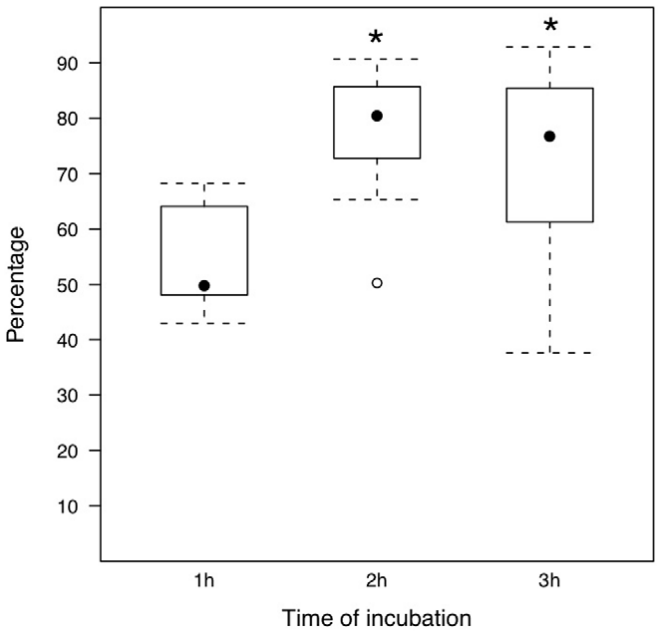
Effect of incubation on percentage of sperm with active caspases. Distribution of the percentage of sperm expressing active caspase-3 and -7 detected in ejaculated sperm after one, two, or three hours of incubation. Values are medians (solid circle) with 25–75% interquartile ranges (box); dotted vertical lines indicate minimum and maximum values; open circle indicate outlier. Values of five replicates. * P<0.05 vs one hour.

To analyze the role of time of incubation on activation of caspases aliquots of ejaculated sperm were incubated for one, two, or three hours before evaluation of active caspases. The percentage of total sperm expressing active caspases increased after two (P = 0.026) or three (P = 0.045) hours of incubation (Fig. 4).

**Figure 5:**
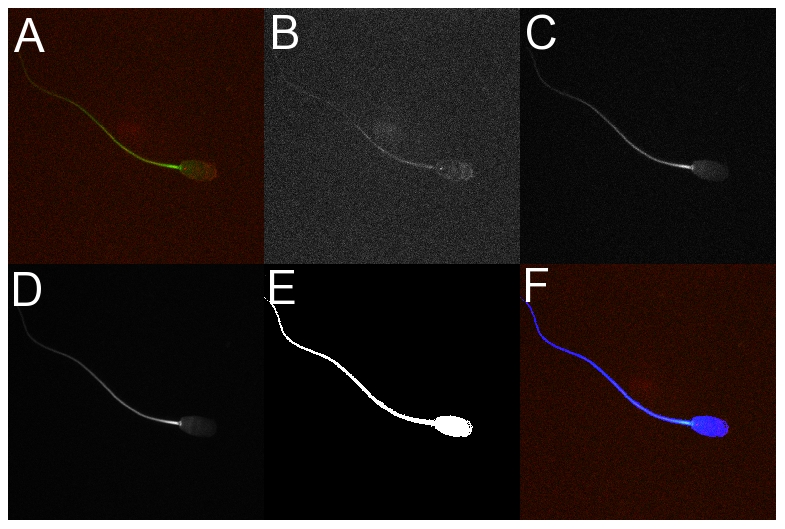
Decomposition of confocal image to analyze pixels forming a sperm cell. Montage of digital images that illustrates the decomposition of a confocal image, the obtention of a mask and the overlay of the mask on channels red and green to exclusively analyze pixels in a sperm cell. A, RGB image when red (active caspases) and green (intracellular esterases) are observed. B, channel red. C, channel, green. D, channel blue. E, mask created from channel blue. F, composite image where mask was merged with channels red and green.

**Figure 6:**
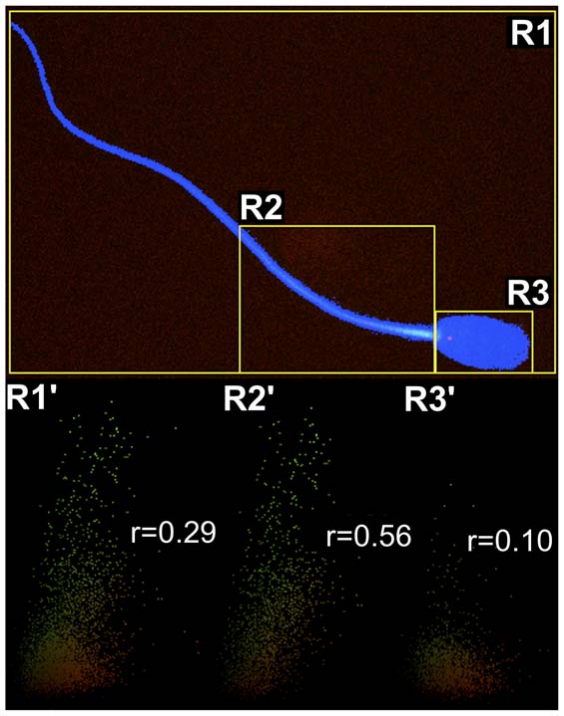
Correlations of active caspases and intracellular esterases in a regionalized sperm cell. Confocal image of a ram sperm coexpressing active caspase-3 and-7 and intracellular esterases. Regions of interest were drawn (yellow rectangles) to represent whole (R1), midpiece (R2) and head sperm (R3) regions, respectively. R1′, R2′ and R3′ show dotplots of values of red versus green pixel values (color points) from R1, R2 and R3 regions respectively (yellowed points indicates co-localized pixels). Text and values in R1′, R2′, and R3′ represents Spearman value for each region. Only pixels conforming the sperm (i. e., inside blue mask) were used in dotplots and included in correlation analysis.

Confocal images of sperm coexpressing active caspases and cytoplasmic esterases were digitally processed to separate red and green channels and to determine threshold pixels of sperm in the blue channel to create a mask before conducting the correlation analysis (Fig. 5). The relationship between the confocal images for red and green pixels is shown in Table 1. A differential relation was observed for whole sperm cells or for different segments. The magnitude of the correlation between red and green pixels of sperm was as follows: midpiece>whole>head (Fig. 6 and Table 1).

**Table 1:** Statistical resume of the Spearman correlation between pixels of channel red (caspase-3 and 7) and channel green (intracellular sterases) for confocal sperm images.

	Spearman correlation		
	Whole sperm	Head sperm	Midpiece sperm
Minimum	0.1400	−0.1400	0.3400
Maximum	0.4900	0.3900	0.7700
Range	0.2500	0.5300	0.4300
Mean	0.3388	0.1112	0.5318
SE	0.0503	0.0816	0.0519

n = 25.

The mean percentages of sperm expressing active caspases from caput, corpus or cauda of epididymis did not differ. However, the percentages of total sperm expressing active caspases in different regions of epididymis were lower than those of the ejaculates.

## Discussion

The presence of active caspases has been demonstrated in the sperm of ovine [22], [8] and other species [23], [9], including humans [5], [6]. For humans, a high percentage of sperm with active caspases has been related with infertility [24]. However, the ejaculates of ovine normally present a high percentage of sperm with active caspases [8]. On other hand, presence of active caspases could correlate negatively [25] or not correlate with motility [8] but not correlate with viability [8]. Recently, using FLICA to detect active caspases, we observed intense staining in cytoplasmic droplets of boar [15] and ram sperm (observations not published), a similar observation was reported early for human sperm [14]. The cytoplasmic droplet is reminiscent of the “cytoplasmic state” of spermatozoa. It is in the middle piece of the sperm where the cytoplasmic droplet leaves the cell to become a residual body fated to be phagocytised by Sertoli cells in the testis [26].

In this work, we observed different sperm subpopulations, one of which presents concomitantly cytoplasmic esterases and active caspases. The fact that sperm subpopulations with different cellular properties such as the presence of cytoplasmic esterases and active caspases are detected in an ejaculate could indicate differences in alteration during transit through the epididymis or alteration during human management. We did not observe changes in the percentage of sperm with active caspases from the caput, corpus, or cauda of the epididymis, but the percentage of ejaculated sperm with active caspases increased after three hours of incubation. The fact that changes in the percentage of sperm with active caspases in the different regions of epididymis were not observed in this work indicates that 1) these molecules are carried in an active state from the time they leave the testis or 2) that conditions of incubation could activate caspases. This supports the suggestion of events of “abortive apoptosis” proposed by Sakkas et al. [13]. Apoptosis can be induced by chemical [7] and physical factors [27]. The increase in the percentage of sperm with active caspases after two or three hours of incubation observed in this work is in conflict with the reports for human [5] and stallion sperm [9]. Possibly the differences in results are due to the species involved or to the medium of incubation; however, at this time is not possibly to clarify this.

For testicular germ cell of rats, it has been demonstrated that some markers of apoptosis are restricted to residual bodies [4], suggesting that the apoptotic machinery moves the germ cells from a cytoplasmic state to a “without cytoplasm state” during differentiation toward mature sperm. It is possible that alterations during spermiogenesis permit sperm with markers of apoptosis to leave the seminiferous epithelium and continue the journey until ejaculated. Related to this, Sakkas et al. [13] coined the term “abortive apoptosis” for those ejaculated sperm that present with molecular apoptosis markers, and Weng et al. [12] suggested that caspases may be sequestered in a region where the mitochondria and remnants of the cytoplasmic droplet could be located in abnormal/or immature sperm. The site where cytoplasmic constituents leave the maturing sperm is precisely the middle piece [26], and in this site an intense FLICA-staining has been observed for human [14] and boar sperm [15]. In this work we demonstrated that a strong relationship exists between the presence of active caspases and cytoplasmic esterases for the middle region of ovine sperm; however, intriguingly, active caspases could be present in other regions of sperm apparently not associated with the cytoplasmic droplet, such as equatorial and apical sections in head or principal section in tail [8]. In head and principal section we do not observed correlation between presence of active caspases and esterases. Whether active caspases presents in ejaculated sperm form a population of cells that has escaped from apoptosis as specified [13], [28] remains to be clarified.

On the other hand, two studies indicated that FLICA inhibitors could associate with no identified target(s) other than cysteine of active caspases, although the expression follows the pattern of apoptosis [29], [30]. In this sense, we have observed in this and other works [8], [15] that intensity of FLICA signals in sperm diminish when a non fluorescent caspase inhibitor is added before FLICA.

For humans a broad range of percentages of sperm with active caspases has been observed when FLICA is used (∼15–80%) [14], [25], and these percentages are related with the origin of the semen samples (donors or patients) and with quality characteristics (normal or abnormal values) [14], [25]. Also the proportion of sperm with active caspases is related with specie [7], [8]. However, sperm with active caspases are a high proportion in ram's [8], and our results are in according with that report.

We do not know why the percentages of of sperm expressing active caspases were high in our study; an explanation to this is related with a possible low specificity of FLICA reagent, however it is discarded due to a increase of sperm with active caspases is observed when an inducer of active caspases is added to incubating sperm; conversely, the percentage of sperm with active caspases do not increase when an inhibitor of active caspases is added to incubating sperm before addition of the inducer of active caspases, as observed in our controls.

In summary, we found that ejaculated sperm form a heterogeneous population that includes subpopulations one of which carries active caspases and cytoplasmic esterases. In sperm marked for active caspases and cytoplasmic esterases, there is a correlation between these two markers in the middle piece. Active caspases are activated during incubation but not during epididymis traverse. It is possible, then, that active caspases are carried in that state from the testis.
